# Lipidomic and transcriptomic profiles provide new insights into the triacylglycerol and glucose handling capacities of the Arctic fox

**DOI:** 10.3389/fvets.2024.1388532

**Published:** 2024-06-26

**Authors:** Yuhang Zhu, Yuan Yuan, Huazhe Si, Songze Li, Fei Zhao, Ruina Mu, Zihan Lin, Xiaoxu Wang, Qiang Qiu, Chao Xu, Lele Ji, Zhipeng Li

**Affiliations:** ^1^College of Animal Science and Technology, Jilin Agricultural University, Changchun, China; ^2^School of Ecology and Environment, Northwestern Polytechnical University, Xi’an, China; ^3^Key Lab of Animal Production, Product Quality and Security, Ministry of Education, Jilin Agricultural University, Changchun, China; ^4^College of Plant Protection, Jilin Agricultural University, Changchun, China; ^5^Department of Special Animal Nutrition and Feed Science, Institute of Special Animal and Plant Sciences, Chinese Academy of Agricultural Sciences, Changchun, China; ^6^National Demonstration Center for Experimental Preclinical Medicine Education, The Fourth Military Medical University, Xi'an, China

**Keywords:** fatty acid oxidation, insulin-like growth factor 1, lipid droplets, uridine, VLDL, glycogen

## Abstract

The Arctic fox (*Vulpes lagopus*) is a species indigenous to the Arctic and has developed unique lipid metabolism, but the mechanisms remain unclear. Here, the significantly increased body weight of Arctic foxes was consistent with the significantly increased serum very-low-density lipoprotein (VLDL), and the 40% crude fat diet further increased the Arctic fox body weight. The enhanced body weight gain stems primarily from increased subcutaneous adipose tissue accumulation. The adipose triacylglycerol and phosphatidylethanolamine were significantly greater in Arctic foxes. The adipose fatty-acid synthase content was significantly lower in Arctic foxes, highlighting the main role of exogenous fatty-acids in fat accumulation. Considering the same diet, liver-derived fat dominates adipose expansion in Arctic foxes. Liver transcriptome analysis revealed greater fat and VLDL synthesis in Arctic foxes, consistent with the greater VLDL. Glucose homeostasis wasn’t impacted in Arctic foxes. And the free fatty-acids in adipose, which promote insulin resistance, also did not differ between groups. However, the hepatic glycogen was greater in Arctic foxes and transcriptome analysis revealed upregulated glycogen synthesis, improving glucose homeostasis. These results suggest that the superior fat accumulation capacity and distinct characteristics of hepatic and adipose lipid and glucose metabolism facilitate glucose homeostasis and massive fat accumulation in Arctic foxes.

## 1 Introduction

The Arctic fox (*Vulpes lagopus*), the smallest nonhibernating carnivore in Arctic regions, has developed notable adaptations to increase its survival rate and withstand extreme winter temperatures (1). These adaptations include a dietary preference for seabirds, eggs and animal carcasses (2), which leads to a significant increase in body mass through the accumulation of adipose tissue during autumn (3). The increased dietary fat consumption is usually associated with nonalcoholic fatty liver disease (NAFLD), diabetes and hyperlipidemia in humans and rodents (4, 5). However, the intake of a diet containing 40% crude fat does not have adverse effects on hepatic fat accumulation or serum triacylglycerol (TAG) levels in the Arctic fox (6). In contrast, the silver fox (*Vulpes vulpes*), a species evolutionary close to the Arctic fox, displays a significantly increased serum TAG level upon consumption of a diet containing 26.11% crude fat in comparison with a 14.71% crude fat diet (7). This stark discrepancy suggests that the Arctic fox possesses unique adaptations for lipid metabolism.

To better understand these advantageous characteristics of the Arctic fox, comparative genomic analyses have been performed and have highlighted several positively selected genes related to lipid metabolism and pyrimidine metabolism (8, 9), identified a single-nucleotide polymorphism of Insulin induced gene 2 (*Insig2*) in the Arctic fox, and increased expression of this gene reduced hepatic lipogenesis in rats (10). Moreover, comparative transcriptomic analysis of the liver, brain and kidney in the Arctic fox revealed that *Gltpd1* and *Akt2*, genes associated with fatty acid metabolism, were under positive selection (11). *Akt2*, in particular, promotes anabolic lipid metabolism in the liver through the insulin signaling pathway (12). These findings collectively underscore the significance of the genetic basis of lipid metabolism in the Arctic fox and the interconnection between fatty acid and glucose metabolism.

The liver is the central organ that controls lipid and glucose homeostasis (13). TAG can be packaged into very-low-density lipoprotein (VLDL) particles and exported from the liver, with increased VLDL assembly ameliorating NAFLD caused by hepatic lipid droplet accumulation (14, 15). Additionally, the liver maintains blood glucose homeostasis through glycogen synthesis and breakdown (16). Moreover, insulin-like growth factor 1 (IGF-1) produced in the liver not only mediates glycogen synthesis (17) but also functions as a critical hormone for fuel metabolism, increasing glucose tolerance and insulin sensitivity in mice (18). Further evidence for the role of IGF-1 in lipid metabolism includes the significant increase in plasma IGF-1 during the period of rapid weight gain in the reindeer (19) and brown bear during the nonhibernation period when fat depots accumulate (20).

**Figure 1:**
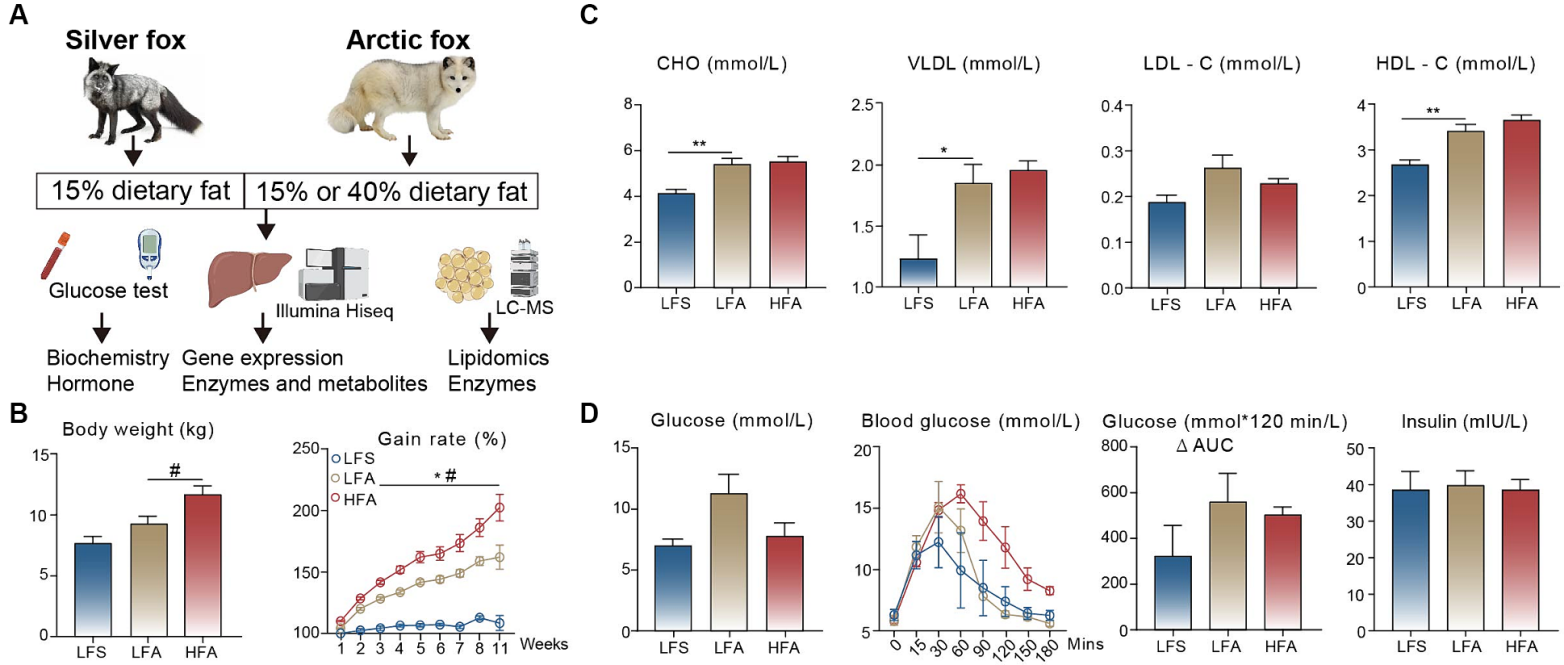
Comparison of serum biochemical analysis and glucose tolerance. **(A)** The experimental design of this study. **(B)** Final body weights and body weight gain rate (% of initial) and **(C)** serum levels of total cholesterol (CHO), very-low-density lipoprotein (VLDL), low-density lipoprotein cholesterol (LDL-C) and high-density lipoprotein cholesterol (HDL-C) among the three groups. The number of animals in the LFS, LFA, and HFA groups were 4, 5, and 5, respectively. **(D)** Assessment of glucose tolerance between arctic fox (*n* = 3) and silver fox (*n* = 3). Results showed as mean ± standard error of the mean (SEM). * indicates significant differences between the LFA group and the LFS group (*p* < 0.05), # indicates significant differences between the HFA group and the LFA group (*p* < 0.05). * *p* < 0.05, ** *p* < 0.01.

In this study, we investigated the body weight gain rate, serum biochemical parameters, and glucose tolerance of the Arctic fox and silver fox. Moreover, we compared the hepatic gene expression profiles using bulk RNA sequencing and further validated these profiles by determining the concentrations of metabolites or proteins. Finally, we determined the differences in lipidomic component and key enzyme concentrations in adipose tissue (Figure 1A).

## 2 Materials and methods

### 2.1 Animals, experimental design, and sample collection

Sixteen healthy male captive Arctic foxes [body weight (BW) = 5.74 ± 0.10 kg] and 7 healthy male captive silver foxes (BW = 7.08 ± 0.43 kg) that were maintained in the research farm of Jilin Agricultural University were included in this study. The Arctic foxes were randomly assigned to either a 15% crude fat diet group (*n* = 8, LFA group) or a 40% crude fat diet group (*n* = 8, HFA group). Silver foxes were fed a 15% crude fat diet (*n* = 7, LFS group). The dietary components and composition are shown in Supplementary Table S1. Each animal was individually housed, and following a one-week adaptation period, then they were fed each diet for 11 weeks, respectively. The body weight was recorded weekly. All animal procedures were approved and authorized by the Animal Ethics Committee of Jilin Agricultural University (No.20210314002) and used the ARRIVE guidelines 2.0 (21).

At the end of the study, blood samples were collected from the hindlimb vein, and centrifuged at 3,500 g for 10 min at 4°C to obtain serum. Animals were then intravenously injected with 5 mL of succinylcholine (0.4 mg/mL), and subcutaneously adipose tissue and liver tissue were collected. Tissue samples were cut into small pieces (approximately 0.5 × 0.5 cm) and immediately frozen in liquid nitrogen for further analysis. Liver samples were also cut into small pieces (1.0 × 1.0 cm) and fixed in 10% neutral buffered formalin for histological assessment.

### 2.2 Glucose homeostasis test

The intraperitoneal glucose tolerance test (IPGTT) was performed according to previous established methods (22). Briefly, 3 animals from each group at 9 weeks were selected to conduct the IPGTT. Following a 14 h overnight fast, animals were received intraperitoneal administration glucose (1 g/kg). Whole blood samples were collected from the hindlimb vein before administration (0 min) and at time points (15, 30, 60, 90, 120, 150, and 180 min) after administration, and blood glucose levels were measured using an Accu-Chek glucometer (Roche Diagnostics Shanghai Ltd., China).

### 2.3 Assessment of metabolites, enzymes, hormones, and liver histology

Serum samples was used to determine the concentrations of glucose, aspartate transaminase (AST), alanine transaminase (ALT), TAG, total cholesterol (CHO), high-density lipoprotein cholesterol (HDL-C) and low-density lipoprotein cholesterol (LDL-C) using the commercial kits (Jiancheng Bioengineering Institute, Nanjing, China) with an automatic biochemical analyzer (Vitalab Selectra E, Vitalab, Dieren, Netherlands). The concentrations of growth hormone (GH), IGF-1, insulin and VLDL in serum were measured using the commercial ELISA kits (MLbio, Shanghai, China).

The liver samples fixed in 10% neutral buffered formalin were embedded in paraffin, cut to 5 μm sections, and stained using H&E (23), and then were visualized using an Olympus AX80 microscope (Olympus Optical, Tokyo, Japan) equipped with Nikon D2X high-resolution camera (Nikon, Tokyo, Japan). The lipid droplet areas were quantified by Image J 1.53a software, and the lipid droplet areas were divided by the area of image to calculate the percentage of lipid droplet areas (24). The concentrations of glucose, TAG, adenosine 3′, 5′-phosphate (cAMP), uridine, glycogen, insulin-like growth factor 1 receptor (IGF1R) and insulin receptor substrate 1 (IRS1) in the liver were measured by the commercial kits (Jiancheng Bioengineering Institute, Nanjing, China) according to the manufacturer’s instructions.

### 2.4 Liver bulk-RNA sequencing and bioinformatics analysis

Total RNA from liver samples was extracted using the RNeasy Mini Kit (QIAGEN, CA, United States). RNA purity, concentration and integrity were determined using a NanoPhotometer^®^ (IMPLEN, CA, United States), an Agilent 2,100 bioanalyzer in combination with the RNA Nano 6,000 assay kit (Agilent Technologies, CA, United States). A total of 1–3 μg of RNA from each sample with an integrity number greater than 7.0 was used to construct the RNA-Seq library using the VAHTS Universal V6 RNA-seq Library Prep Kit for Illumina^®^. The cDNA concentration of constructed library was measured using the Qubit^®^ RNA Assay Kit in Qubit^®^ 3.0. Each library was then sequenced on a Novaseq 6000 platform, producing 150 bp paired-end reads.

Trimmomatic (25) was used to remove low quality and adapter sequences, and HISAT2 was employed to align the remaining clean reads to the reference genomes of the Arctic fox and silver fox, respectively (8, 26, 27). Gene expression level was calculated using the raw count data. Differentially expressed genes (DEGs) were determined using the DeSeq2 package (28) based on the threshold |log_2_ FC| > 0.5 and Benjamini-Hochberg adjusted *p* value <0.05. The weighted gene coexpression network analysis (WGCNA) was conducted to explore the correlation between DEGs and serum biochemical indices using the WGCNA package (29). The clusterProfiler package was utilized to conduct Gene Ontology (GO) term and Kyoto Encyclopedia of Gene and Genomics (KEGG) pathway enrichment analyses (30).

### 2.5 Adipose lipidomic analysis and enzyme examination

A total of 20 mg adipose tissue from each animal in the LFA and LFS groups was dissolved in 400 μL water, vortexed for 60 s, and homogenized at 45 Hz for 4 min, and sonicated for 5 min in ice-water bath. The homogenization and sonication circle were repeated for 3 times. A 20 μL portion of the homogenate was mixed with 180 μL water, and then 480 μL extract solution (MTBE: MeOH = 5:1) containing internal standard (Supplementary Table S2) was added. After vortexing for 60 s, the samples were sonicated for 10 min in ice-water bath. The samples were then centrifuged at 3,000 rpm for 15 min at 4°C. A 250 μL supernatant was transferred to a fresh tube. The remaining sample was mixed with 250 μL of MTBE, followed with vortex, sonication and centrifugation, and another 250 μL supernatant was collected. The supernatants were combined and dried in a vacuum concentrator at 37°C and were reconstituted in 200 μL resuspension buffer (DCM: MeOH: H_2_O = 60: 30: 4.5). The samples were vortexed for 30 s and sonicated for 10 min in ice-water bath. The mixture was then centrifuged at 12,000 rpm for 15 min at 4°C, and 35 μL of supernatant was transferred to a fresh glass vial for LC/MS analysis.

The ultra-high-performance liquid chromatography (UHPLC) separation was performed using a SCIEX ExionLC series UHPLC System. The mobile phase A consisted of 40% water, 60% acetonitrile, and 10 mmol/L ammonium formate. The mobile phase B consisted of 10% acetonitrile and 90% isopropanol, and 10 mmol/L ammonium formate. The column temperature was set to 45°C, while the auto-sampler temperature was set to 6°C. The injection volume was 2 μL. The Biobud-v2.1.4.1 Software (Biotree Biotech Co., Ltd., Shanghai, China) was used to quantify the metabolites. The absolute content of individuals lipids corresponding to the internal standard was calculated based on peak area and the actual concentration of the identical lipid class internal standard. Principal component analysis (PCA) was applied to reveal the differences of lipids. The value of variable importance in the projection (VIP) of the first principal component in orthogonal projections to latent structures discriminant analysis (OPLS-DA) was determined. Metabolites with VIP > 1 and a Benjamini-Hochberg adjusted *p* value <0.05 (student’s *t*-test) were considered as significantly changed metabolites (31).

The concentrations of LPCAT4 and FAS were measured by commercial kits from Jiancheng Bioengineering Institute (Nanjing, China) and BiotechPack (Beijing, China), respectively, according to the manufacturer’s instructions.

### 2.6 Statistical analysis

One-way ANOVA was utilized to determine the statistical significance of final body weights, serum biochemical indices and hormones, areas under curves of IPGTT, liver lipid droplets areas, metabolites and proteins among the LFA, HFA, and LFS groups. The unpaired *t*-test was performed to identify the statistical significance of adipose metabolites and proteins between the LFS and LFA groups. The statistical analysis was performed using Graphpad Prism (version 9.0.0, GraphPad Software Inc., San Diego, CA, United States). All *p* value < 0.05 were considered to indicate statistical significance.

## 3 Results

### 3.1 Comparison of the growth, biochemical indices, and IPGTT among the three groups

We monitored body weight of Arctic and silver foxes fed a 15% crude fat diet and found that the body weight gain rate of Arctic foxes was significantly greater than that of silver foxes over the 11-week monitoring period. In addition, consumption of a 40% crude fat diet led to further increases in the body weight and the body weight gain rate of Arctic foxes (Figure 1B). Consequently, the thickness of subcutaneous adipose tissue in Arctic foxes was approximately 3 times greater than that in silver foxes (Supplementary Figure S1), indicating that the weight gain observed in Arctic foxes was due primarily to fat deposition. We measured serum biochemical parameters and found that the serum concentrations of CHO, VLDL and HDL-C in Arctic foxes were significantly greater than those in silver foxes fed a 15% crude fat diet (*p* < 0.05; Figure 1C). Notably, no differences were observed in these serum biochemical parameters between Arctic foxes fed a 15% crude fat diet and those fed a 40% crude fat diet, indicating that Arctic foxes can maintain normal blood lipid levels on a 40% crude fat diet. The serum glucose and insulin levels did not differ significantly among the three experimental groups (Figure 1D). In addition, we conducted an IPGTT and found that the glucose metabolism capacity did not differ significantly among the groups (Figure 1D), suggesting that liver function and glucose homeostasis remained largely unaffected.

### 3.2 Significant differences in gene expression, the concentrations of protein and metabolite in the liver

**Figure 2:**
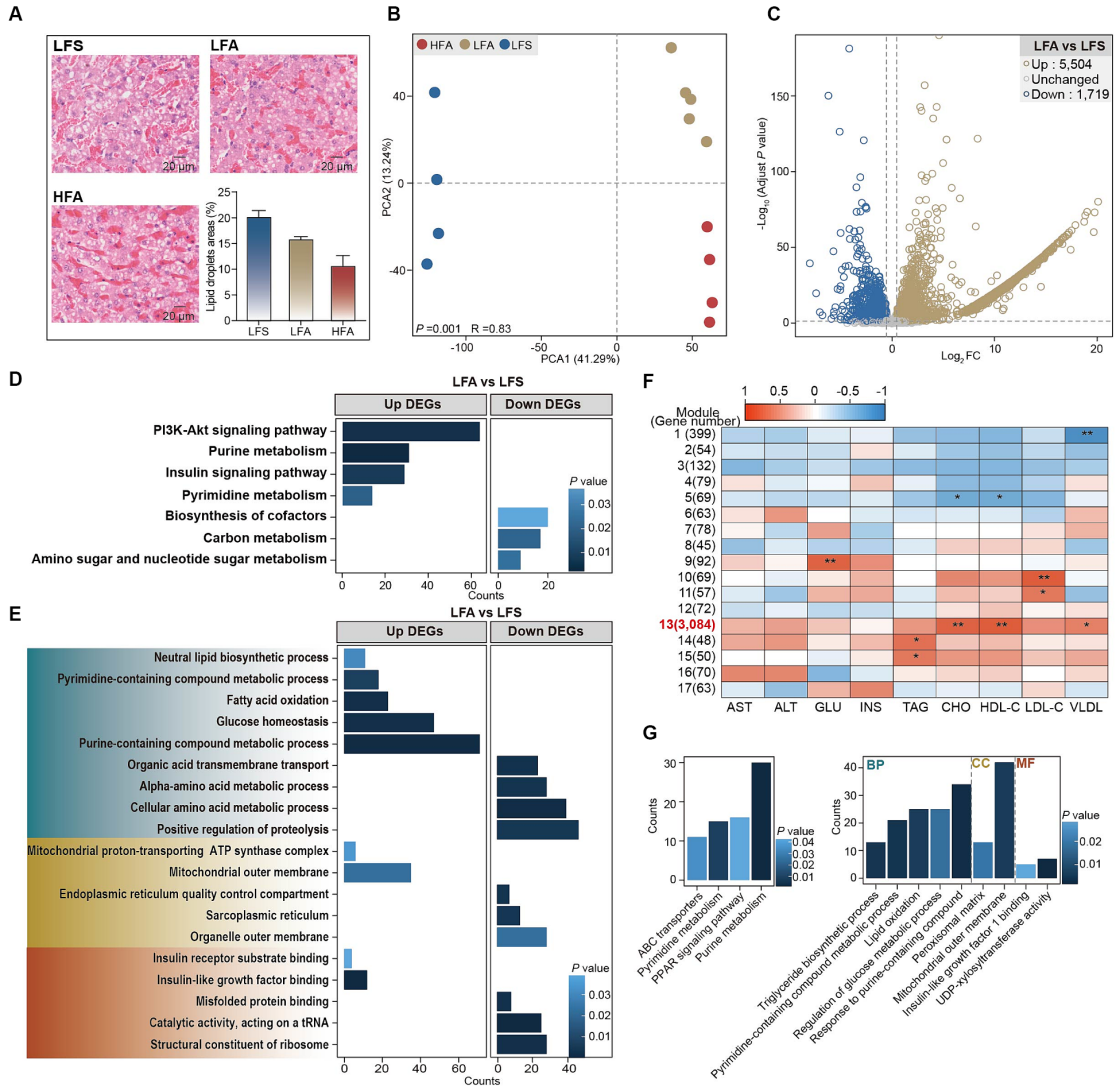
The results of histological analysis and transcriptome analysis of liver. **(A)** Liver H&E staining (scale bar, 20 μm) and lipid droplet area in liver (*n* = 3 in each group). Results showed as mean ± SEM. **(B)** The PCA plot based on the gene count to reveal the expression profiles in liver among 3 groups. **(C)** Volcano plot to show the up- and down- differentially expressed genes (DEGs) in the comparison between LFA versus LFS. **(D)** Kyoto Encyclopedia of Gene and Genomics (KEGG) enrichment analysis of DEGs. **(E)** Gene Ontology (GO) enrichment analysis of DEGs. GO terms in green, yellow and red regions belonging to biological process, cell component and molecular function, respectively. **(F)** Correlation between the identified weighted gene coexpression network analysis (WGCNA) modules and the serum biochemical parameters. **(G)** KEGG and GO enrichment analysis of genes in the module 13. Up, significantly upregulated DEGs; Down, significantly downregulated DEGs. * *p* < 0.05, ** *p* < 0.01. GLU, glucose; INS, insulin.

Histopathological analysis indicated that even upon consumption of a 40% crude fat diet, the lipid droplet area within the liver remained lower in Arctic foxes than in silver foxes (Figure 2A). Accordingly, consumption of a 40% crude fat diet did not increase the hepatic TAG content in the Arctic fox, nor did it result in elevated serum levels of AST and ALT (Supplementary Figure S2A). Transcriptomic analysis revealed significant differences in global gene expression profiles between LFA and LFS groups as well as between the LFA and HFA groups (*p* < 0.01; Figure 2B). This analysis led to the identification of DEGs in the following comparisons: LFA versus LFS and HFA versus LFA. A total of 5,504 DEGs were significantly upregulated and 1,719 DEGs were downregulated between the LFA and LFS groups (Figure 2C). In contrast, the comparison between the HFA and LFA groups revealed 30 DEGs, 15 of which were upregulated and 15 of which were downregulated (Supplementary Figure S2B), suggesting that consumption of a 40% crude fat diet does not induce substantial alterations in the liver function of Arctic foxes.

The upregulated DEGs in LFA versus LFS were significantly enriched in the PI3K-Akt signaling pathway (*Igf1*, *Igf1r*, *Irs1*), purine metabolism pathway (*Nme2*, *Cant1*, and *Adcy6*), insulin signaling pathway and pyrimidine metabolism pathway (*Nme3*, *Cant1*, and *Nt5e*) (Figure 2D; Supplementary Figure S2C). These DEGs were also significantly enriched in the following biological process terms: purine-containing compound metabolic process, glucose homeostasis, fatty acid oxidation (*Acox1*, *Acadm*, *Ppara*), pyrimidine-containing compound metabolic process and neutral lipid biosynthetic process (*Gpat3*, *Lpin3*, *Dgat1*, Figure 2E; Supplementary Figure S2C). In addition, insulin-like growth factor binding and insulin receptor substrate binding were noted as enriched molecular function terms (Figure 2E). The downregulated DEGs were enriched in the biosynthesis of cofactors, carbon metabolism, and amino sugar and nucleotide sugar metabolism pathways (Figure 2D); the biological process terms positive regulation of proteolysis and cellular amino acid metabolic process; the cellular component terms organelle outer membrane and sarcoplasmic reticulum; and the molecular function terms structural constituent of ribosome and catalytic activity (Figure 2E).

**Figure 3:**
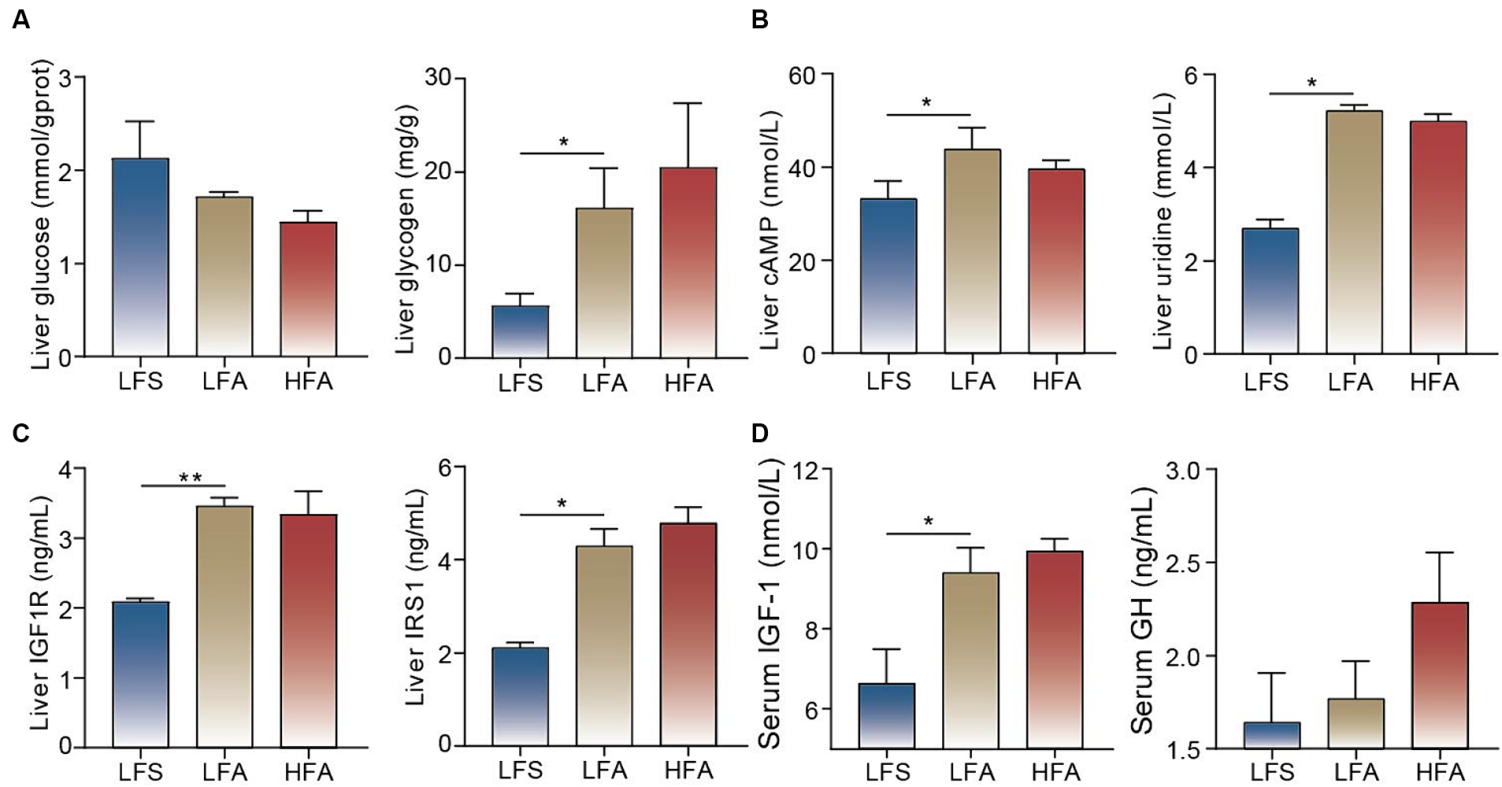
Measurement of key metabolites and hormones. **(A)** Glucose and glycogen concentrations in liver. **(B)** cAMP and uridine concentrations in liver. **(C)** Insulin like growth factor 1 receptor (IGF1R) and insulin receptor substrate 1 (IRS1) concentrations in liver among the three groups. **(D)** The concentrations of insulin-like growth factor 1 (IGF-1) and growth hormone (GH) in serum among the three groups. Results showed as mean ± SEM. * *p* < 0.05, ** *p* < 0.01.

WGCNA was used to explore the associations between the DEGs and serum biochemical parameters, and 17 modules containing between 45 and 3,084 genes per module were identified (Figure 2F). Module 13 showed a significant positive correlation with the serum concentrations of CHO, HDL-C and VLDL (Figure 2F). The genes within module 13 were enriched in pathways such as ABC transporters, pyrimidine metabolism, PPAR signaling pathway and purine metabolism (Figure 2G). Triglyceride biosynthetic process, pyrimidine-containing compound metabolic process, lipid oxidation, regulation of glucose metabolic process, and response to purine-containing compound were identified as enriched biological process terms. Insulin-like growth factor 1 binding and UDP-xylosyltransferase activity were identified as enriched molecular function terms (Figure 2G). Taken together, these findings suggest that pyrimidine, purine, and glucose metabolism, along with insulin-like growth factor and insulin receptor responses, are likely enhanced in the liver of Arctic foxes. Further analysis revealed significantly greater hepatic concentrations of glycogen, cAMP, uridine, IGF1R and IRS1 in Arctic foxes than in silver foxes (*p* < 0.05); in contrast, the hepatic concentration of glucose was lower in Arctic foxes than in silver foxes, although the difference was nonsignificant (Figures 3A–C). Additionally, the serum concentration of IGF-1 was significantly greater in Arctic foxes than in silver foxes, although the serum concentration of GH was not significantly different between Arctic and silver foxes (Figure 3D).

### 3.3 Significantly increased TAG and PE concentrations in the adipose tissue of Arctic foxes

**Figure 4:**
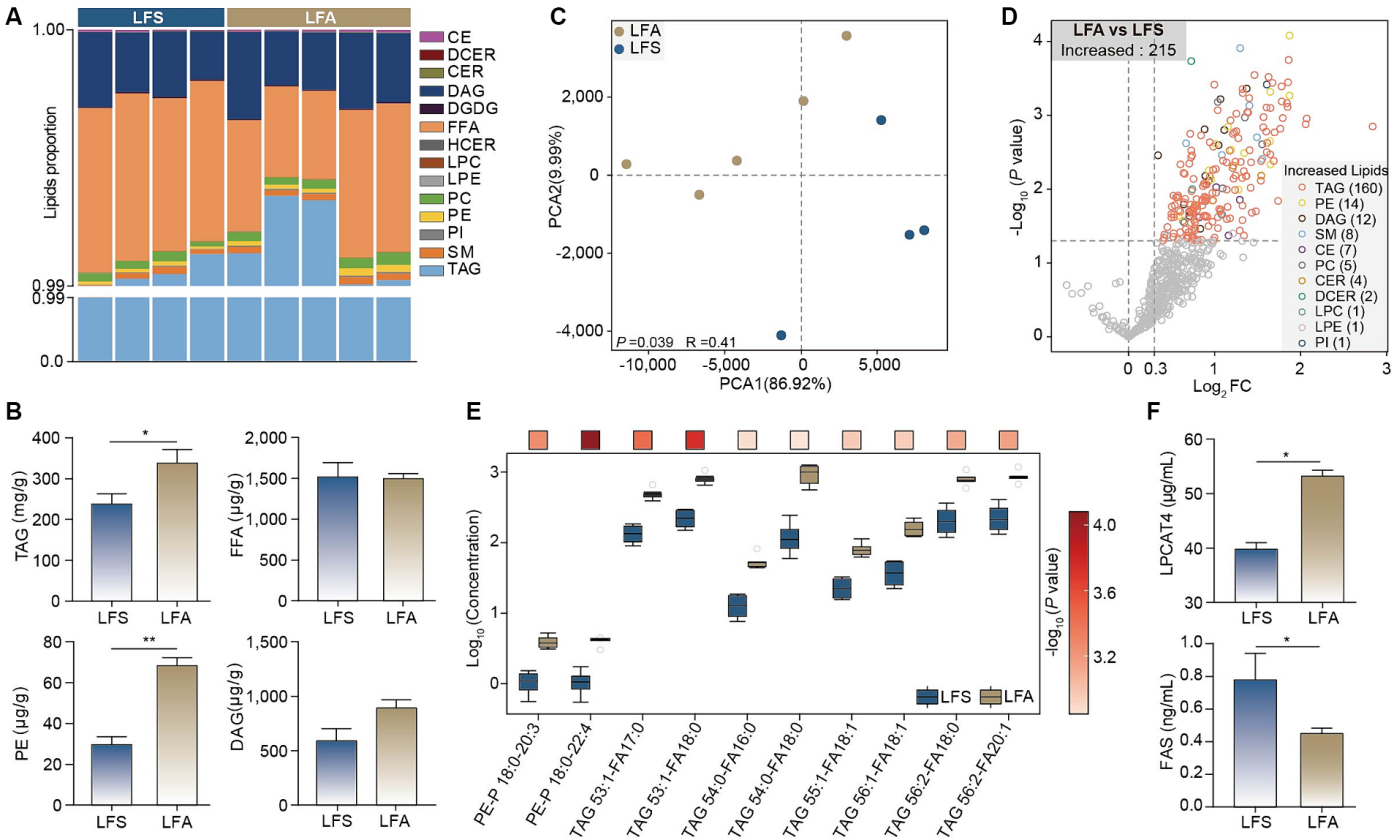
Lipidomic analysis and enzymes of adipose tissue. **(A)** The composition and proportion of the identified lipids in adipose tissue. **(B)** Differences in total concentrations of TAG, FFA, PE and DAG in adipose tissue. **(C,D)** PCA and volcano plot revealing the differences of lipid components between arctic fox and silver fox. **(E)** The top 10 different lipids in adipose tissue between arctic fox and silver fox. The color of the square indicates the significance of the different lipids from maximum (red) to minimum. **(F)** The concentrations of LPCAT4 and FAS in adipose tissue. Results showed as mean ± SEM. PE, phosphatidylethanolamines; DAG, diacylglycerols; SM, sphingomyelins; CE, cholesterol esters; PC, phosphatidylcholines; CER, ceramides; DCER, dihydroceramides; LPC, lysophosphatidylcholine; PI, phosphatidylglycerol; LPE, lysophosphatidylethanolamines; FFA, free fatty acids. * *p* < 0.05, ** *p* < 0.01.

Given the significant changes in body mass, serum cholesterol and liver transcriptome profiles in Arctic foxes (LFA group) compared with silver foxes (LFS group), we explored the lipid composition and concentration in adipose tissue and identified 443 TAGs, 46 diacylglycerols (DAGs) and 26 phosphatidylethanolamines (PEs) (Supplementary Figure S3). TAGs constituted more than 99% of the lipid content in all the samples (Figure 4A). The total concentrations of TAG (*p* < 0.05) and PE (*p* < 0.01) in Arctic foxes were significantly greater than those in silver foxes, while the total concentration of free fatty acids (FFAs) was not significantly different (Figure 4B). The PCA results revealed that the lipid profiles were significantly different between the LFA and LFS groups (*p* < 0.05; Figure 4C). The abundances of 215 lipids were significantly increased in Arctic foxes compared with silver foxes, and the numbers of significantly increased TAGs, PEs and DAGs were 160, 14 and 12, respectively (Figure 4D; Supplementary Tables S3, S4). Further comparison revealed that 8 of the 10 lipids with the greatest log_2_ fold change (FC) values were TAGs and the other 2 were PEs (Figure 4E), indicating likely increases in TAG and PE synthesis. We thus measured the concentrations of LPCAT4 and FAS that catalyze the synthesis of PEs and fatty acids in adipose tissue. The concentration of LPCAT4 (*p* < 0.01) in Arctic foxes was significantly greater than that in silver foxes, while the concentration of FAS in Arctic foxes was significantly less than that in silver foxes (*p* < 0.05) (Figure 4F), suggesting the PE synthesis is increased and fatty acid synthesis is decreased in Arctic foxes.

## 4 Discussion

Our results revealed greater subcutaneous adipose deposition and body weight gain in Arctic foxes when fed a 15% or 40% crude fat diet than in silver foxes when fed a 15% crude fat diet. Previous studies revealed dramatic fat deposition in the Arctic fox and brown bear in autumn (3, 32). These findings confirmed the notable ability for fat accumulation in Arctic animals. We also found that the TAG and PE concentrations in the adipose tissue of Arctic foxes were significantly greater than those in the adipose tissue of silver foxes. This finding is consistent with the observation that the TAG and PE concentrations in adipose tissue of brown bears were greater during hibernation than during the active state (33). When the concentration of TAGs between the leaflets of the endoplasmic reticulum bilayer increases, TAGs coalesce and eventually form lipid droplets (34). It has also been reported that PE is involved in lipid droplet formation and stability (35). Moreover, the concentration of LPCAT4, the key enzyme in PE biosynthesis (36), was also significantly greater in Arctic foxes than in silver foxes. These results suggest that increased PE biosynthesis likely contributes to fat accumulation in Arctic foxes by promoting TAG storage in lipid droplets. We also found that the serum IGF-1 level was significantly higher in Arctic foxes than in silver foxes. This finding is in line with previous findings that plasma IGF-1 is increased in the reindeer and brown bear during the accumulation of fat depots (19, 20). IGF-1 can also induce lipid synthesis through the PI3K-Akt signaling pathway (37, 38). Therefore, the increased amount of IGF-1 is likely responsible for TAG and PE synthesis in the adipose tissue of Arctic foxes. Hepatic synthesis and release of IGF-1 are primarily affected by GH (39). However, the serum GH level was not significantly different between Arctic foxes and silver foxes. The expression of *Igf1r* in the liver regulates the serum IGF-1 level through a negative feedback mechanism (40). Our results showed that *Igf1r* was significantly upregulated in Arctic foxes and that the IGF1R concentration in the liver was significantly greater in Arctic foxes than in silver foxes. Therefore, the increased IGF-1 production in Arctic foxes is likely associated with IGF1R-mediated negative feedback.

Interestingly, we found that the FFA content in adipose tissue, the insulin level in serum and glucose tolerance were not significantly different between Arctic foxes and silver foxes. The brown bear also remains insulin sensitive during the fat accumulation period (32). Our results thus indicated the key role of FFA metabolism homeostasis in maintaining insulin sensitivity in the Arctic fox. However, the concentration of the crucial enzyme of *de novo* lipogenesis, FAS (41), was significantly lower in Arctic foxes than in silver foxes, leading to decreased *de novo* lipogenesis. Dietary and liver-derived FFAs can also be transported into adipose tissue. FFAs can be esterified by diglyceride acyltransferase on the surface of lipid droplets and stored in lipid droplets in the form of TAGs (42). The TAG concentration in adipose tissue was significantly greater in Arctic foxes than in silver foxes; considering the consumption of the same diet and the decreased *de novo* lipogenesis in Arctic foxes, liver-derived FFAs could be the main source for TAG accumulation in Arctic foxes. Therefore, the esterification of FFAs to TAGs likely plays a role in maintaining FFA homeostasis, and liver-derived FFAs can dominate fat accumulation in the adipose tissue of Arctic foxes. Moreover, a previous study demonstrated that IGF-1 can promote adipogenesis (43), resulting in increased lipid storage and decreased insulin resistance (44). Therefore, the increased IGF-1 expression in Arctic foxes also likely promotes lipid accumulation in adipose tissue and prevents insulin resistance.

Lipid droplets are ubiquitous intracellular storage organelles specialized for the storage of excess energy in the form of neutral lipids. Surprisingly, we found that the lipid droplet area and TAG concentration in the livers of Arctic foxes were less than those in the livers of silver foxes. However, body weight gain is usually associated with an increased risk of abnormal lipid accumulation in the liver (45). Liver transcriptome analysis revealed greater differences in lipid metabolism-related genes than in other types of genes between Arctic foxes and silver foxes, and the genes upregulated in Arctic foxes were enriched in the neutral lipid biosynthesis process and pyrimidine metabolism, which involves *Dgat1* and *Nt5e*. Moreover, the genes positively correlated with the serum concentrations of CHO, HDL-C and VLDL were significantly enriched in the triglyceride biosynthetic process and pyrimidine metabolism. These findings are consistent with those of a comparative genomic analysis revealing that the positively selected genes in the Arctic fox were also significantly enriched for lipid metabolism and pyrimidine metabolism (8). The lipid metabolism and pyrimidine metabolism pathways have been reported to play important roles in hepatic lipid metabolism (46). *Dgat1* encodes a crucial enzyme involved in TAG synthesis (47), and *Nt5e* is essential for uridine synthesis during pyrimidine metabolism (48). These results indicate the crucial roles of lipid and pyrimidine metabolism in hepatic lipid homeostasis in Arctic foxes. The uridine concentration in the liver and the VLDL concentration in the serum of Arctic foxes were significantly greater than those in silver foxes. TAGs synthesized in the endoplasmic reticulum can be used for the synthesis of lipid droplets or VLDL (49). An increase in VLDL was reported to ameliorate lipid accumulation in liver (14). Previous studies revealed a role for uridine in inhibiting lipid droplet synthesis in the liver (50). *Dgat1* is also involved in VLDL synthesis and can increase the plasma VLDL concentration, while *Dgat2* (downregulated in Arctic foxes) is associated with liver steatosis (51). These results revealed that the Arctic fox may prevent abnormal lipid droplet accumulation by increasing uridine and VLDL synthesis.

Purine metabolism and fatty acid oxidation, which involve *Adcy6*, *Ppara, Acadm*, and *Acox1*, were also identified as significantly enriched in the liver of Arctic foxes. The proliferator-activated receptor alpha (PPARα) protein encoded by *Ppara* promotes the expression of *Acadm* and *Acox1*, inducing fatty acid oxidation in mitochondria and peroxisomes (52, 53). cAMP, a product of purine metabolism, can activate PPARα through the cAMP/PKA pathway (54), and *Adcy6* is the crucial gene for cAMP production (55). Moreover, the liver cAMP concentration was significantly greater in Arctic foxes than in silver foxes. These results reveal that fatty acid oxidation is likely promoted in the Arctic fox through the cAMP/PKA pathway. The PI3K-AKT signaling pathway, insulin signaling pathway and the expression of *Slc2a4*, which encodes glucose transporter type 4 (GLUT4), were significantly upregulated in Arctic foxes. The PI3K-AKT signaling pathway and insulin signaling pathway are important for glucose homeostasis (56) and GLUT4 transports glucose into the liver (57). However, the liver glycogen concentration in Arctic foxes was significantly greater than that in silver foxes, while the glucose concentration was not significantly different between the two species. The key gene involved in glycogen synthesis, *Gys1* (58), was significantly upregulated in Arctic foxes. In addition, IGF-1 can promote *Gys1* expression through the PI3K-AKT signaling pathway (59). These results indicate that the Arctic fox may maintain glucose homeostasis by increasing liver glycogen synthesis.

**Figure 5:**
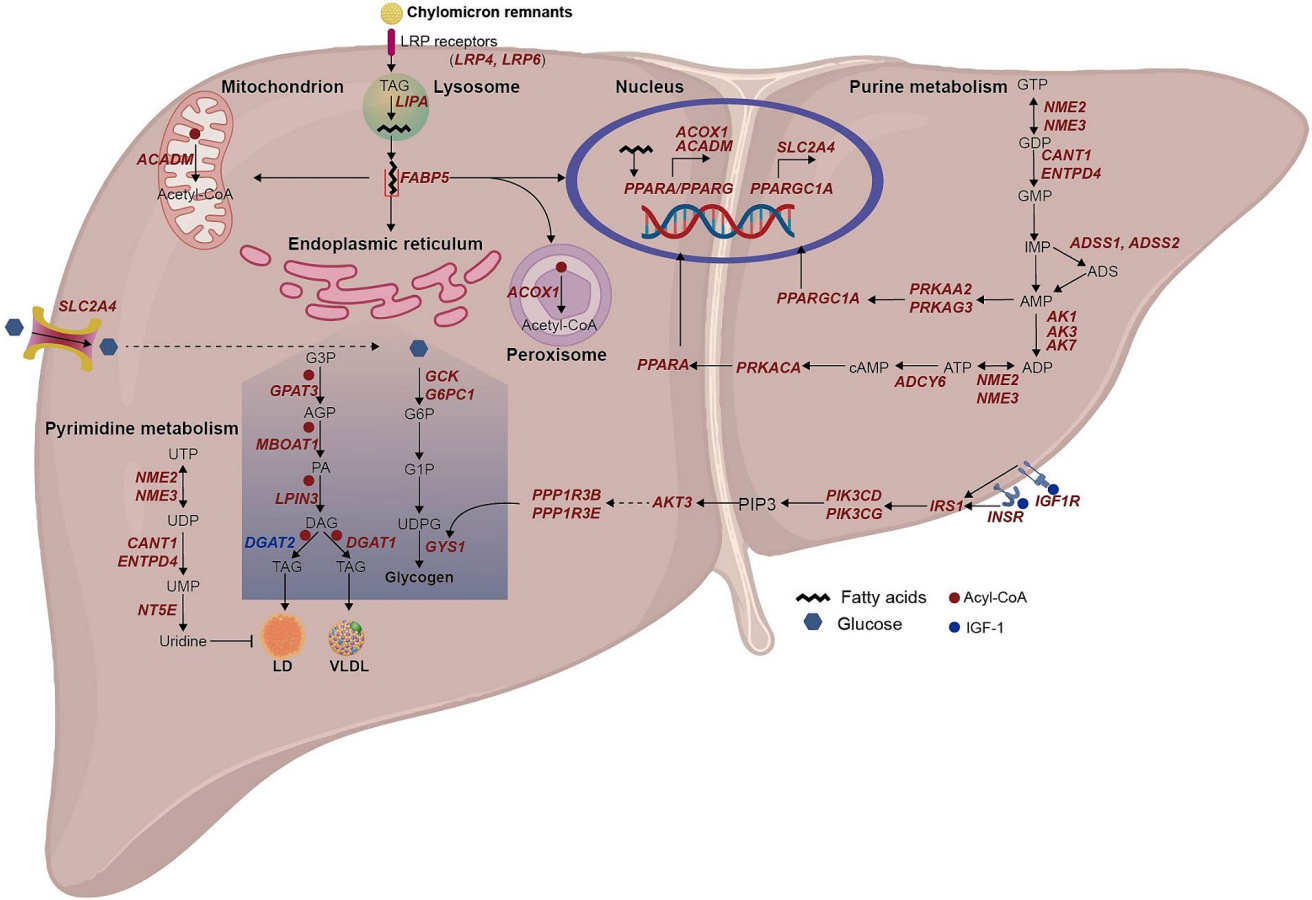
Schematic summary of lipid and glucose metabolism in hepatocyte of arctic fox. The italic red and blue texts indicate up-and down-regulated DEGs in LFA group compared to LFS group. We manually reconstructed the metabolic pathways in liver of Arctic foxes based on the significantly enriched GO terms and KEGG pathways, and the significantly different metabolites, enzymes and hormones. Then the figure was drawn using Adobe Illustrator. UTP, uridine triphosphate; UDP, uridine 5′-diphosphate; UMP, uridine monophosphate; G3P, glycerol 3-phosphate; AGP, 1-Acyl-glycerol 3-phosphate; PA, phosphatidate; DAG, diacylglycerols; TAG, triacylglycerol; LD, lipid droplet; G6P, glucose 6-phosphate; G1P, glucose 1-phosphate; UDPG, uracil-diphosphate glucose; GTP, guanosine 5′-triphosphate; GDP, guanosine 5′-diphosphate; GMP, guanosine 5′-phosphate; IMP, inosine monophosphate; ADS, adenylosuccinate; AMP, adenosine 5′-monophosphate; ADP, adenosine 5′-diphosphate; ATP, adenosine 5′-triphosphate; cAMP, adenosine 3′, 5′-phosphate; PIP3, Phosphatidylinositol-3,4,5-triphosphate.

In this study, we confirmed that the Arctic fox adapts to its diet through unique alterations in lipid and glucose metabolism in liver tissue (Figure 5). The Arctic fox can increase TAG storage from liver-derived fat by increasing PE synthesis in adipose tissue. Additionally, it can prevent aberrant lipid droplet accumulation in the liver by increasing uridine and VLDL synthesis and cAMP/PKA pathway-mediated fatty acid oxidation. Moreover, the Arctic fox can maintain glucose homeostasis by increasing liver glycogen synthesis.

## Data availability statement

The data presented in the study are deposited in the NCBI repository, accession number PRJNA1028137.

## Ethics statement

The animal study was approved by Ethical Committee of Jilin Agricultural University. The study was conducted in accordance with the local legislation and institutional requirements.

## Author contributions

YZ: Formal analysis, Investigation, Writing – original draft, Software, Visualization. YY: Formal analysis, Data curation, Software, Writing – review & editing. HS: Investigation, Writing – review & editing. SL: Investigation, Writing – review & editing. FZ: Investigation, Writing – review & editing. RM: Investigation, Writing – review & editing. ZhL: Investigation, Writing – review & editing. XW: Investigation, Writing – review & editing. QQ: Writing – review & editing. CX: Writing – review & editing. LJ: Conceptualization, Methodology, Supervision, Writing – review & editing. ZpL: Conceptualization, Formal analysis, Funding acquisition, Methodology, Project administration, Supervision, Writing – review & editing.

## Supplementary material

Supplementary Figure S1 The thickness of subcutaneous adipose tissue among the LFS, LFA and HFA groups.

Supplementary Figure S2 Comparison of metabolites, enzymes and gene expression between Arctic foxes and silver foxes. **(A)** The concentrations of liver TAG and serum AST and ALT among the LFS, LFA and HFA groups. **(B)** Volcano plot to show the up- and down- differentially expressed genes (DEGs) in liver between HFA and LFA. **(C)** Heatmap showing the DEGs in liver between Arctic foxes and silver foxes, which are involved in glucose and lipid metabolism. TAG: triacylglycerol; AST: aspartate transaminase; ALT: alanine transaminase.

Supplementary Figure S3 The composition of lipids in the subcutaneous adipose tissue based on the lipidomic analysis. The percentage in the brackets of pie chart means the proportion of lipids, and the numbers in the brackets of legend means the numbers of lipids. CE: cholesterol esters; CER: ceramides; DAG: diacylglycerols; DCER: dihydroceramides; DGDG: digalactosyldiacylglycerol; FFA: free fatty acids; HCER: hexosylceramides; LPC: lysophosphatidylcholines; LPE: lysophosphatidylethanolamines; PC: phosphatidylcholines; PE: phosphatidylethanolamines; PI: phosphatidylinositol; SM: sphingomyelins; TAG: triacylglycerols.

Supplementary TABLE S1 Ingredients of diets (DM basis).

Supplementary TABLE S2 The information of internal standard in lipidomic analysis.

Supplementary TABLE S3 The identified lipids in the subcutaneous adipose tissue of the LFA and LFS groups.

Supplementary TABLE S4 Significantly different lipids between the LFA and LFS groups.
