# Supplementary figures and images for: Lipidomic and transcriptomic profiles provide new insights into the triacylglycerol and glucose handling capacities of the Arctic fox
Source: Front Vet Sci. 2024 Jun 26;11:1388532. doi: 10.3389/fvets.2024.1388532 (PMC11233799; doi:10.3389/fvets.2024.1388532)

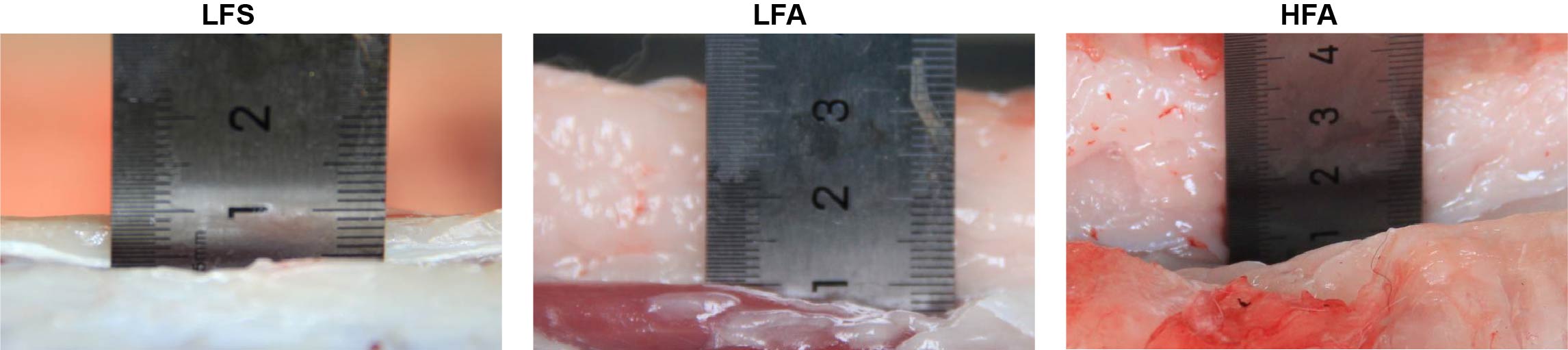

Supplement: Supplementary Figure S1 — The thickness of subcutaneous adipose tissue among the LFS, LFA and HFA groups. [file Image_1.JPEG]

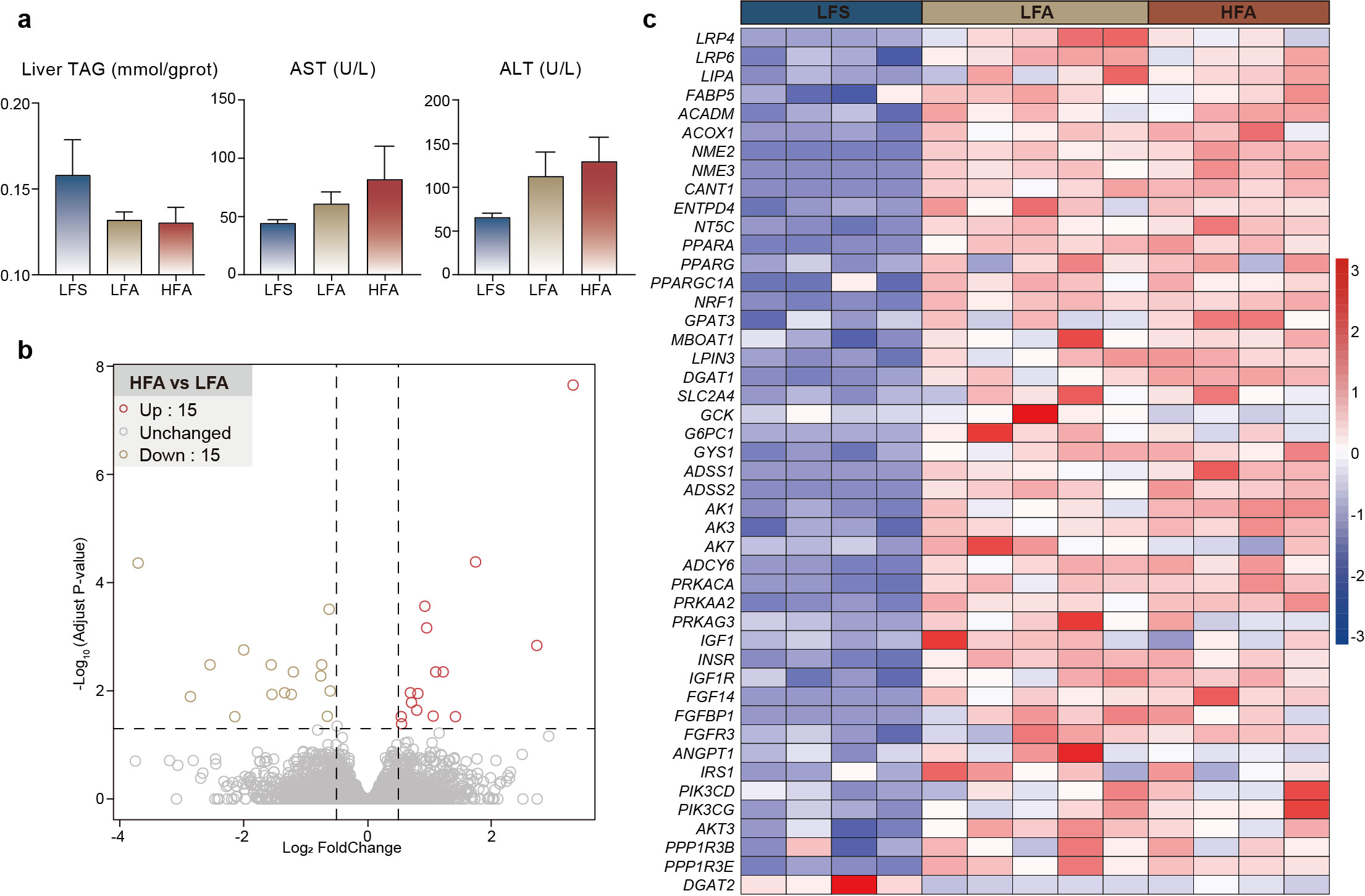

Supplement: Supplementary Figure S2 — Comparison of metabolites, enzymes and gene expression between Arctic foxes and silver foxes. (A) The concentrations of liver TAG and serum AST and ALT among the LFS, LFA and HFA groups. (B) Volcano plot to show the up- and down- differentially expressed genes (DEGs) in liver between HFA and LFA. (C) Heatmap showing the DEGs in liver between Arctic foxes and silver foxes, which are involved in glucose and lipid metabolism. TAG: triacylglycerol; AST: aspartate transaminase; ALT: alanine transaminase. [file Image_2.JPEG]

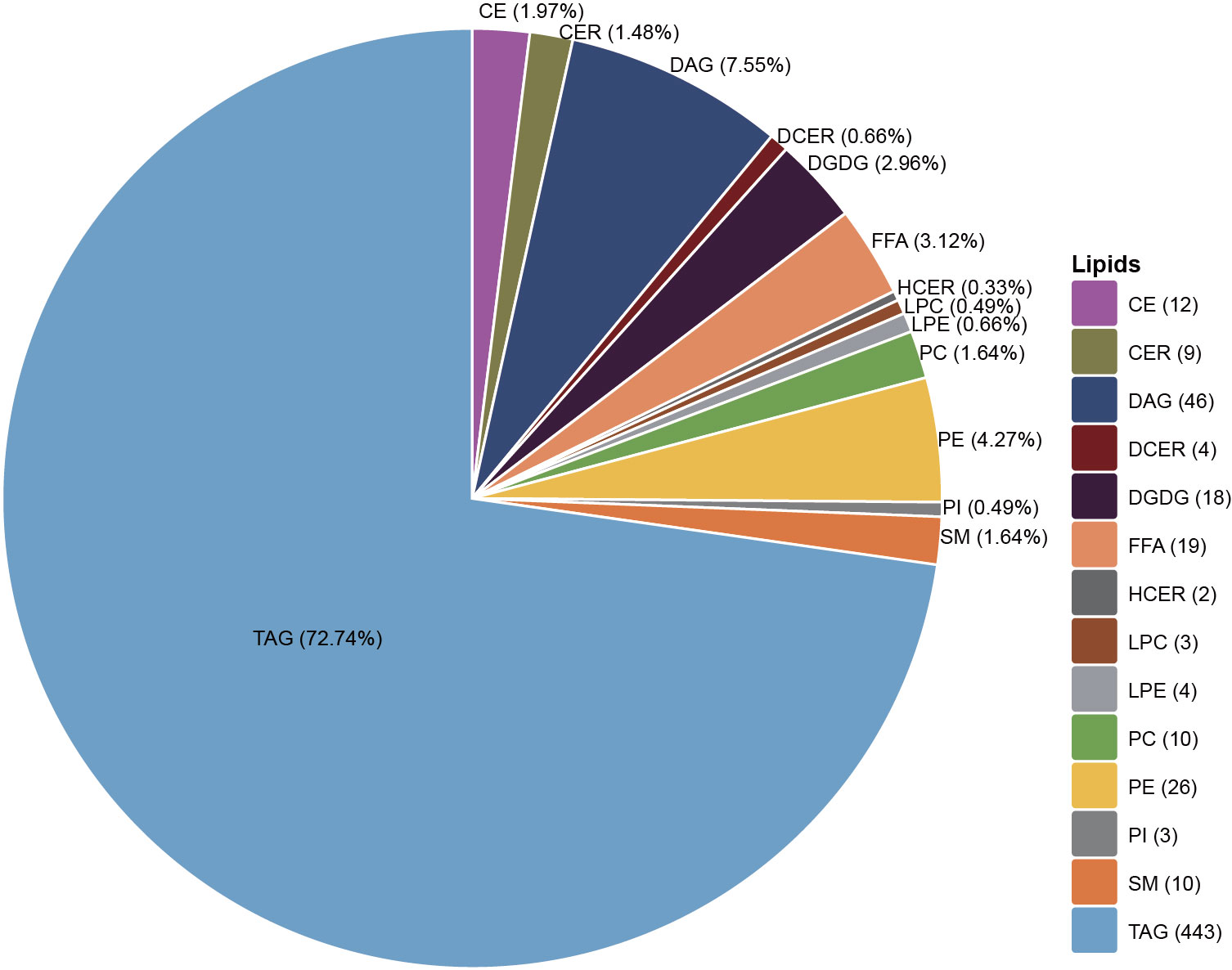

Supplement: Supplementary Figure S3 — The composition of lipids in the subcutaneous adipose tissue based on the lipidomic analysis. The percentage in the brackets of pie chart means the proportion of lipids, and the numbers in the brackets of legend means the numbers of lipids. CE: cholesterol esters; CER: ceramides; DAG: diacylglycerols; DCER: dihydroceramides; DGDG: digalactosyldiacylglycerol; FFA: free fatty acids; HCER: hexosylceramides; LPC: lysophosphatidylcholines; LPE: lysophosphatidylethanolamines; PC: phosphatidylcholines; PE: phosphatidylethanolamines; PI: phosphatidylinositol; SM: sphingomyelins; TAG: triacylglycerols. [file Image_3.JPEG]
